# Lifestyle, Modifiable Behavioral Factors, and Biomarker Profiles in Uterine Lesions

**DOI:** 10.3390/healthcare14020231

**Published:** 2026-01-16

**Authors:** Anna Mihaylova, Antoniya Yaneva, Angelina Mollova-Kyosebekirova, Ekaterina Uchikova, Desislava Bakova, Mariya Koleva-Ivanova, Mariana Parahuleva, Nikoleta Parahuleva

**Affiliations:** 1 Department of Healthcare Management, Faculty of Public Health, Medical University of Plovdiv, 4000 Plovdiv, Bulgaria; desislava.bakova@mu-plovdiv.bg; 2 Department of Medical Informatics, Biostatistics and E-Learning, Faculty of Public Health, Medical University of Plovdiv, 4002 Plovdiv, Bulgaria; antoniya.yaneva@mu-plovdiv.bg; 3 Department of General and Clinical Pathology, Medical University of Plovdiv, 4002 Plovdiv, Bulgaria; angelina.mollova@mu-plovdiv.bg (A.M.-K.); mariya.koleva@mu-plovdiv.bg (M.K.-I.); 4 Department of Obstetrics and Gynecology, Faculty of Medicine, Medical University of Plovdiv, 4000 Plovdiv, Bulgaria; ekaterina.uchikova@mu-plovdiv.bg (E.U.); nikoleta.parahuleva@mu-plovdiv.bg (N.P.); 5 Universitätsklinikum Gießen und Marburg Standort Marburg, 35043 Marburg, Germany; mariana.parahuleva@prof-parahuleva.de

**Keywords:** endometrial cancer, endometrial intraepithelial neoplasia (EIN), leiomyoma, body mass index (BMI), physical activity, behavioral risk factors, lifestyle, health literacy, prevention, quality of life

## Abstract

**Background**: Precursor endometrial lesions and endometrial cancer are strongly influenced by lifestyle-related risk factors, including obesity, low physical activity, and unfavorable dietary patterns. Identifying these factors is essential for early prevention and for improving health literacy among women. **Objective**: The objective of this study was to evaluate the influence of modifiable lifestyle factors on the likelihood of developing EIN and endometrial cancer in comparison with leiomyoma. **Materials and Methods**: A cross-sectional analytical study was conducted among 50 women, divided into three groups: leiomyoma (n = 20), EIN (n = 15), and endometrial cancer (n = 15). BMI, physical activity, dietary habits, sleep duration, stress levels, and smoking status were assessed. Statistical analysis included the Kruskal–Wallis test, correlation analysis, and logistic regression. **Results**: BMI was identified as an independent predictor of EIN/EC (OR = 1.29; *p* = 0.015). Women with EIN/EC demonstrated significantly lower levels of physical activity (*p* = 0.018). A clustering of behavioral risks was observed: higher BMI was associated with higher stress and shorter sleep duration. **Conclusions**: Modifiable lifestyle factors play a key role in the development of precursor and malignant endometrial conditions. Targeted interventions focusing on weight management, increased physical activity, and improved health literacy may reduce risk and improve quality of life among peri- and postmenopausal women.

## 1. Introduction

Endometrial disorders, including leiomyomas, precursor endometrial lesions (atypical hyperplasia/endometrial intraepithelial neoplasia, EIN), and endometrial cancer (EC), represent a significant medical and public health concern, particularly among peri- and postmenopausal women. Endometrial cancer is the most common malignant tumor of the uterus and ranks among the leading oncological diseases in women in industrialized countries [1]. Its development typically involves a transition from hormonally mediated endometrial proliferation to precancerous lesions and subsequent neoplastic transformation [2].

Accumulated evidence highlights the significant role of modifiable lifestyle-related risk factors such as excess body weight, unhealthy dietary patterns, and low levels of physical activity in the pathogenesis of endometrial cancer [3,4]. Obesity is recognized as one of the strongest predictors, increasing the risk of EC between two- and five-fold depending on the degree of elevated BMI [5]. This association is largely attributed to increased peripheral aromatization of androgens in adipose tissue, leading to chronic estrogenic stimulation of the endometrium in the absence of adequate progesterone regulation [6]. Additionally, obesity is frequently associated with insulin resistance, systemic inflammation, and oxidative stress, which further enhance carcinogenic potential [7].

In contrast, regular physical activity has been identified as a protective factor that not only supports weight regulation but also improves hormonal balance, reduces the secretion of pro-inflammatory cytokines, and enhances insulin sensitivity [8]. Conversely, unfavorable dietary patterns characterized by high intake of saturated fats, refined carbohydrates, and ultra-processed foods have been associated with increased risk of endometrial hyperplasia and malignant progression [9].

Additional factors such as disturbed sleep, psychological stress, and smoking are also recognized as components of a complex behavioral profile influencing hormonal regulation, appetite, and metabolic processes [10]. The presence of a family history of cancers within the Lynch spectrum further increases risk, underscoring the interaction between genetic predisposition and environmental/lifestyle factors [11].

Against this background, investigating the influence of lifestyle and behavioral patterns on the development of precursor and malignant endometrial disease is crucial for early prevention, for enhancing health literacy, and for improving health-related quality of life.

Beyond biological mechanisms, an important aspect of understanding endometrial disease is the interplay between behavioral, socioeconomic, and cultural factors shaping women’s health behavior. Numerous studies indicate that low health literacy is directly associated with delayed diagnosis, reduced participation in preventive programs, and limited awareness of the relationship between lifestyle factors and disease risk [12,13]. In the context of endometrial disease, this is particularly relevant, as early symptoms (such as irregular or postmenopausal bleeding) are often underestimated or attributed to “normal hormonal changes”, which may delay seeking medical care.

From a public health perspective, endometrial cancer is a condition in which a substantial proportion of risk is potentially preventable through targeted lifestyle modifications [14]. Interventions focusing on weight control, increased physical activity, improved diet quality, and reduction in alcohol and tobacco use can decrease the prevalence of metabolic and endocrine disturbances that contribute to the development of endometrial lesions. Health promotion programs, including support groups, individualized counseling, digital mobile applications, and community-based interventions, have demonstrated effectiveness in sustaining behavioral change [15].

It is also essential to emphasize the role of quality of life (QoL), which is a key priority in contemporary gynecology and oncology. Women with excess body weight and low physical activity levels frequently experience fatigue, depressive symptoms, and chronic stress, which perpetuate a cycle of unhealthy behavioral choices [16]. Therefore, prevention-oriented interventions should be psychologically supportive rather than purely informational.

At the policy level, international recommendations underscore the importance of a multidisciplinary approach involving healthcare institutions, primary care providers, educational systems, media, and local communities to promote healthy behaviors and encourage regular preventive screenings [17]. Thus, the study of behavioral and social factors is not only a clinical necessity but also a foundation for effective primary and secondary prevention strategies.

## 2. Materials and Methods

### 2.1. Study Design

An observational, comparative, analytical study with a cross-sectional design was conducted. The aim was to evaluate the influence of lifestyle-related risk factors on the likelihood of developing precursor endometrial lesions and endometrial cancer in comparison to a benign condition (leiomyoma).

### 2.2. Study Population

A total of 50 women were included, distributed into three diagnostic groups according to histopathological conclusions: Leiomyoma (n = 20); Endometrial intraepithelial neoplasia/atypical hyperplasia (EIN) (n = 15); Endometrial cancer (n = 15).

**Inclusion criteria:** patients were selected based on the presence of a histologically confirmed diagnosis.

**Exclusion criteria:** pregnancy, active oncological diseases outside the uterus, prior hysterectomy, severe cognitive impairment preventing participation.

### 2.3. Data Collection

Data was collected through:

**Medical documentation (Medical history)**—age, anthropometric parameters, reproductive history, diagnosis.

**Clinical and laboratory indicators related to metabolic, inflammatory, and hormonal status.** Due to variability in available laboratory data across patients, biomarkers were analyzed categorically (normal/elevated/missing data). The following biomarkers were assessed:

- Insulin resistance—recorded presence of diagnosis or laboratory indication of “elevated insulin” or “elevated HOMA-IR”;
- Lipid profile—(LDL↑, HDL↓, TG↑);
- Chronic inflammation—presence of elevated hs-CRP;
- Inflammatory cytokines—documented elevated IL-6 or TNF-α when available;
- Oxidative stress—documented elevated MDA when present in the medical record;
- Hormonal profile—relatively elevated estradiol levels and/or low progesterone/estrogen ratio.

The complete numerical laboratory values were not available for all participants, biomarkers were analyzed categorically (normal/elevated/missing). This approach ensured comparability across the dataset but may have resulted in some loss of information due to reduced granularity.

Biomarkers were categorized using standard clinical thresholds: HOMA-IR > 2.5 (elevated insulin resistance), hs-CRP ≥ 3 mg/L (elevated), IL-6 > 7 pg/mL, TNF-α > 8 pg/mL, LDL-C ≥ 3.4 mmol/L, triglycerides ≥ 1.7 mmol/L, and MDA above the laboratory reference range. Values below these thresholds were classified as normal.

**A structured questionnaire** composed of items adapted from validated international instruments was used to assess lifestyle-related factors. The lifestyle questionnaire included adapted items derived from the IPAQ-SF, MEDAS, PSS, and PSQI domains. These constructs were assessed using single-item or simplified categorical measures. Cultural and linguistic adaptation involved expert review and pilot testing to ensure clarity and contextual appropriateness. The exact wording of all questionnaire items used in the study is provided in Supplementary File S1. Physical activity was evaluated using a shortened and category-based format derived from the International Physical Activity Questionnaire—Short Form (IPAQ-SF), which enabled classification of participants into high (>150 min/week), moderate (75–150 min/week), and low (<75 min/week) activity groups [18]. Dietary habits were assessed using selected items adapted from the Mediterranean Diet Adherence Screener (MEDAS-14), resulting in a dietary index score, where higher values reflected healthier dietary patterns [19].

Perceived stress was evaluated using key items conceptually derived from the Perceived Stress Scale (PSS-10), scored on a 5-point Likert scale (1 = very low stress; 5 = very high stress) [20]. Sleep duration (hours per night) was self-reported in alignment with standard approaches used in the Pittsburgh Sleep Quality Index (PSQI) [21]. Smoking status (never/former/current) and alcohol intake (units/week) were recorded as categorical variables. Body Mass Index (BMI) was calculated and classified according to the World Health Organization criteria.

The simplified lifestyle questionnaire included core domains from the IPAQ-SF (activity categories), MEDAS (dietary score components), PSS (single perceived stress item), and PSQI (sleep duration item). Scores were derived by summing the categorical responses according to predefined criteria described in Supplementary File S1.

### 2.4. Statistical Analysis

As the study was exploratory and based on all consecutively available women with histologically confirmed diagnoses during the study period, a formal a priori sample size or power calculation was not performed. The sample, therefore, reflects the maximum feasible number of eligible patients within the defined timeframe, rather than a predefined target derived from power estimation.

Statistical processing was performed using: descriptive statistics, χ^2^ test to compare distributions, Kruskal–Wallis test to compare BMI between groups, Pearson correlation analysis, and logistic regression to evaluate the independent influence of risk factors. Statistical significance was set at *p* < 0.05.

The multivariable logistic regression models included BMI, physical activity level, dietary index, sleep duration, perceived stress score, and smoking status. These variables were prespecified based on established clinical relevance. In addition, variables with a univariate association with the outcome at *p* < 0.20 were retained for multivariable modeling.

Multicollinearity among the included lifestyle variables was evaluated using Variance Inflation Factors (VIFs). All VIF values were below 3, which was considered acceptable and indicative of no substantial multicollinearity.

### 2.5. Ethical Considerations

The study was conducted in accordance with institutional ethical guidelines and approved by the Ethics Committee of the Medical University of Plovdiv (Protocol 3/28 October 2024), adhering to the principles of the Declaration of Helsinki. All participants provided written informed consent for the use of their data and materials in this research.

## 3. Results

A total of 50 women were included in the study, divided into three groups based on histological diagnosis: leiomyoma (n = 20), EIN/atypical hyperplasia (n = 15), and endometrial cancer (n = 15). The mean age of the participants was 52 ± 9 years, with no statistically significant difference between groups (*p* > 0.05).

Baseline demographic and reproductive characteristics are presented in Table 1. Women with EC were older compared with those with leiomyoma and EIN, although the difference did not reach statistical significance (*p* = 0.175). BMI was higher in the EIN/EC groups compared with the leiomyoma group (*p* = 0.051). No significant differences were observed in age at menarche, menopausal status, or parity.

Lifestyle factors are summarized in Table 2. Physical activity differed significantly between groups (*p* = 0.018), with a higher proportion of women in the EC group reporting high activity levels, and a greater proportion of women in the EIN group reporting low activity. Sleep duration also differed significantly across groups (*p* = 0.021). No significant group differences were observed for alcohol intake, smoking status, perceived stress, or dietary index scores.

### 3.1. Body Mass Index and Dietary Habits

The mean BMI was higher in the EIN and cancer groups compared to the leiomyoma group (30.8 vs. 27.6 kg/m^2^), with the difference approaching statistical significance (Kruskal–Wallis χ^2^ = 5.96; *p* = 0.051). The distribution of BMI categories showed a higher proportion of women with obesity (≥30 kg/m^2^) in the EIN and cancer groups. The mean dietary index was lower in the EIN/EC groups compared to the leiomyoma group, suggesting a less favorable dietary pattern.

### 3.2. Physical Activity

Levels of physical activity differed significantly between the groups (χ^2^ = 11.86; *p* = 0.018). Women with EIN and endometrial cancer more frequently reported low physical activity (<75 min/week) compared to women with leiomyoma, among whom moderate to high activity predominated.

### 3.3. Correlation Profile

The correlation analysis demonstrated:

- A positive relationship between BMI and stress level (r ≈ 0.30);
- A negative relationship between BMI and sleep duration (r ≈ −0.26);
- A negative relationship between dietary index and alcohol consumption (r ≈ −0.34);
- A positive relationship between physical activity and dietary index (r ≈ 0.29).

These associations support the presence of a cluster of unhealthy behavioral patterns.

### 3.4. Logistic Regression

Logistic regression analyses are presented in Table 3. In univariate models, BMI was significantly associated with the combined EIN/EC outcome (OR = 1.23; 95% CI 1.04–1.45; *p =* 0.016). In the fully adjusted multivariable model, BMI remained a significant predictor (AOR = 1.29; 95% CI 1.06–1.58; *p =* 0.012), whereas physical activity, dietary index, sleep duration, stress, and smoking status were not independently associated with the outcome.

**Model A:** (EIN/EC) vs. Leiomyoma

Higher BMI was identified as an independent predictor of the presence of precursor or malignant endometrial changes: OR = 1.29 per one BMI unit increase (95% CI 1.05–1.57; *p* = 0.015).

Other variables (physical activity, dietary index, smoking, sleep, stress) showed trends in the expected direction but did not reach statistical significance with the current sample size.

**Model B:** Endometrial cancer vs. (Leiomyoma + EIN)

No independent predictor reached *p* < 0.05, but higher BMI and lower physical activity showed a positive direction of effect, suggesting probable clinical relevance, limited by the small sample size.

Multicollinearity diagnostics showed acceptable VIF values (<3) for all variables, indicating that the lifestyle factors included in the models did not exhibit problematic collinearity.

Missing data patterns were examined prior to analysis. Missing laboratory values primarily reflected variability in the clinical diagnostic work-up and were treated as missing at random (MAR). Missingness in lifestyle variables was minimal (<5%). A complete-case sensitivity analysis was performed, and the direction of associations remained consistent with the main analyses.

### 3.5. Biomarkers Related to Metabolic and Inflammatory Status

In a subgroup of participants (n = 38) for whom laboratory results were available, the following trends were identified:

- Mean fasting insulin and HOMA-IR levels were higher in the EIN/EC groups compared to the leiomyoma group (HOMA-IR: 2.8 vs. 1.9).
- Levels of hs-CRP (high-sensitivity C-reactive protein) were higher in the EIN/EC groups, indicating low-grade chronic inflammation.
- IL-6 and TNF-α levels showed a tendency toward elevation in the EC group compared to leiomyoma.
- Markers of oxidative stress (e.g., MDA—malondialdehyde) were elevated in the EC group.
- Patients with higher BMI exhibited elevated estrogen levels in the absence of proportional increases in progesterone.

These results support the role of insulin resistance, inflammation, and estrogen-dependent proliferation in the pathogenesis of endometrial lesions.

### 3.6. Biomarkers (Categorical Analysis)

Biomarker distributions for the subgroup with available laboratory data are shown in Table 4. A higher proportion of women with EIN/EC exhibited elevated insulin resistance, hs-CRP, IL-6/TNF-α, and MDA levels compared with the leiomyoma group. Hormonal imbalance (elevated estrogen–progesterone risk profile) was also more frequent among EIN/EC cases.

- The frequency of elevated markers of insulin resistance (insulin/HOMA-IR) was higher in the EIN/EC groups compared to the leiomyoma group (47% vs. 20%).
- Elevated hs-CRP values were identified in 42% of women with EIN/EC compared to 15% of women with leiomyoma.
- Elevated IL-6/TNF-α occurred only in patients with EIN/EC (total of 23%), while no abnormalities were observed in the leiomyoma group.

These trends are consistent with higher BMI and lower physical activity levels in the same groups, highlighting the relationship between metabolic environment, inflammation, and endometrial proliferation.

## 4. Discussion

The findings of this study indicate notable differences in modifiable behavioral factors across women with leiomyoma, EIN, and EC. The lower levels of physical activity observed among women with EIN and EC, compared with those with leiomyoma, are consistent with epidemiological evidence showing that insufficient physical activity is often accompanied by less favorable metabolic and inflammatory profiles [7,8]. Such patterns may contribute to a physiological milieu associated with endometrial proliferation, although the cross-sectional design does not allow for conclusions regarding temporal or causal pathways.

One of the central observations was that BMI emerged as the only independent factor associated with EIN/EC in multivariable analysis. This aligns with clinical guidelines and meta-analytic data identifying elevated BMI as one of the strongest established risk factors for endometrial cancer [3,4]. Mechanistic models described in prior literature suggest that adiposity is linked with increased peripheral estrogen production in adipose tissue and reduced progesterone balance, creating conditions under which endometrial proliferation may occur [5]. Although our results cannot determine directionality, they are consistent with these established associations.

The study also identified clustering of behavioral factors, with higher BMI frequently accompanied by higher perceived stress and shorter sleep duration. Previous research indicates that inadequate sleep and chronic stress may influence hormonal regulation (e.g., cortisol, leptin) and metabolic functioning [10,22]. These patterns suggest that comprehensive behavioral approaches—addressing diet, sleep, stress, and physical activity simultaneously—may be more effective than targeting a single factor alone.

Although the comparison between EIN/EC did not yield statistically significant independent predictors, the observed trends of higher BMI and lower activity among women with EC are consistent with broader research. The modest sample size likely limited statistical power in subgroup comparisons, which is a common constraint in exploratory studies of this scale.

The observed lower physical activity and shorter sleep duration among women with EIN/EC suggest potentially modifiable behavioral targets. These domains may warrant greater emphasis in preventive counseling and health promotion efforts, although longitudinal studies are needed to evaluate their impact on risk trajectories.

From a clinical perspective, the findings underscore the potential utility of incorporating lifestyle and metabolic assessments into routine gynecologic evaluation, particularly for peri- and postmenopausal women. Interventions aimed at supporting sustained behavior change, such as structured education, counseling, and digital tools—may hold value for improving overall health trajectories, although future longitudinal studies are needed to establish their impact on disease progression.

The inclusion of metabolic and inflammatory biomarkers adds further context to the behavioral patterns observed. Elevated insulin resistance, hs-CRP, and IL-6 among women with EIN/EC are consistent with prior work linking excess adiposity to chronic low-grade inflammation and altered endocrine signaling [6,7]. Similarly, elevated cytokines such as IL-6 and TNF-α have been associated with oxidative stress and genomic instability in the endometrium. Regular physical activity has been shown in previous studies to mitigate these processes by improving insulin sensitivity and reducing inflammation [8], which may help explain the distribution patterns seen in our cohort, though causal inference cannot be made.

Overall, the biomarker profiles observed in this study complement the behavioral findings and reinforce the relevance of integrated lifestyle strategies focused on weight management, physical activity, dietary habits, and stress reduction. Further research with larger and longitudinally followed cohorts is needed to clarify temporal relationships and refine prevention-oriented recommendations.

## 5. Strengths of the Study

The present study has several key strengths that enhance its scientific and practical relevance. First, it integrates a comprehensive analysis of modifiable behavioral factors (dietary patterns, physical activity, sleep, stress levels) that are directly related to the risk and progression of endometrial lesions. This approach allows the identification of specific behavioral profiles associated with a higher risk of transition to precursor and malignant conditions, which is essential for practical prevention strategies.

Second, the inclusion of objective clinical and laboratory data, such as markers of insulin resistance, systemic inflammation, and hormonal status (although analyzed categorically), enriches the analysis by linking behavioral patterns to underlying metabolic and hormonal mechanisms. This enables the interpretation of findings within the context of well-established pathophysiological pathways connecting obesity, inflammation, and endometrial proliferation.

Third, the study employs a comparative design involving three clinical groups (leiomyoma, EIN, and endometrial cancer), which allows the identification of trends related to the transition from benign to precancerous and malignant states. This design contributes to a better understanding of progression dynamics and emphasizes the role of lifestyle factors at different stages of disease development.

Finally, the study has high public health significance, as its findings provide a basis for developing practical, targeted health promotion programs, including weight management, improved dietary counseling, encouragement of physical activity, and psychological support. These implications have direct applicability in clinical practice, primary prevention, and educational interventions aimed at improving health literacy among peri- and postmenopausal women.

## 6. Limitations

This study has several methodological limitations that should be considered when interpreting the findings. The sample size is relatively moderate, which may reduce the statistical power of multivariate analyses. Additionally, the lack of longitudinal data limits the ability to draw prognostic conclusions regarding disease progression.

In addition, no formal a priori power calculation was conducted, and the modest sample size may have limited the ability to detect some clinically relevant associations, particularly for lifestyle factors other than BMI, increasing the risk of type II error. As a result, the present findings should be interpreted as exploratory and hypothesis-generating.

The categorical treatment of biomarkers represents an additional limitation. Due to variability in the availability of numerical laboratory values in the medical records, biomarkers could not be analyzed on a continuous scale, which may introduce misclassification and reduce the precision of effect estimates.

The presence of missing laboratory data represents an additional limitation. Although missingness appeared to follow a missing-at-random pattern, the reduced sample size for biomarker analyses may have affected the precision of estimates.

Several methodological considerations merit attention. Lifestyle variables were self-reported, which may introduce some degree of reporting variability. The study was conducted at a single institution, and therefore the sample may not fully represent broader populations. Socioeconomic factors (e.g., education, income), which often influence lifestyle behaviors, were not collected. Although reproductive parameters such as menopausal status, parity, and hormonal therapy use were recorded, they were not included in multivariable models given the modest sample size. In addition, the categorical classification of certain biomarkers (normal/elevated/missing) may have led to loss of granularity, particularly for values near clinical cutoffs.

As a cross-sectional study, the results reflect associations at a single time point and cannot distinguish whether lifestyle patterns or biomarker alterations precede, result from, or co-evolve with endometrial changes.

## 7. Summary

BMI was identified as an independent predictor of the presence of EIN/EC compared to leiomyoma (OR ≈ 1.29 per BMI unit; *p* = 0.015). Physical activity differed significantly between diagnostic groups (*p* = 0.018), with lower activity associated with less favorable conditions. The correlation profile supports multicomponent lifestyle interventions.

## 8. Conclusions

The present study demonstrates that modifiable lifestyle factors play a substantial role in the development of precursor and malignant endometrial changes. Elevated BMI was identified as an independent predictor of EIN and EC, emphasizing the importance of metabolic and hormonal mechanisms associated with obesity. Low physical activity was significantly more common among patients with precancerous and malignant lesions, confirming its role as a protective factor.

The observed clustering of behavioral risk factors (higher BMI, lower dietary index, shorter sleep duration, and higher stress levels) highlights the need for multimodal interventions that target the overall lifestyle rather than isolated habits.

The findings underscore the importance of improving health literacy and implementing early preventive measures, particularly among peri- and postmenopausal women. Interventions focusing on weight optimization, regular physical activity, stress management, improved sleep, and routine gynecological examinations could substantially reduce risk and improve quality of life.

The observed trends are consistent with current evidence and emphasize the need for early behavioral interventions and educational programs as key components of strategies for preventing precursor and malignant endometrial conditions.

## 9. Recommendations for Improving Health Literacy

1. **Weight control:** Individualized goals for BMI and waist circumference; dietitian-supported nutritional counseling.
2. **Physical activity:** ≥150 min/week of moderate activity; behavioral “fractionation” into 10–15 min sessions when needed.
3. **Nutrition:** Increased intake of vegetables and whole grains; limitation of ultra-processed foods and sugar-sweetened beverages.
4. **Sleep and stress:** Sleep hygiene strategies; relaxation and breathing techniques; support for chronic stress management.
5. **Tobacco and alcohol:** Support for smoking cessation; limitation of alcohol consumption.
6. **Targeted communication:** Short, accessible visual educational materials for women at increased risk (including postmenopausal women, BMI > 30, and low physical activity levels).

## 10. Perspectives

The present study highlights the need for a deeper investigation into the interaction between modifiable behavioral factors and the biological mechanisms involved in the development of precursor and malignant endometrial conditions. Future research should include:

1. **Larger and more representative cohorts** to allow more precise evaluation of the individual and combined contributions of specific risk factors.
2. **Prospective (longitudinal) studies** monitoring changes in lifestyle behaviors and their influence on progression from benign to precancerous and malignant states.
3. **Integration of biomarkers**, including hormonal profiles, markers of insulin resistance, inflammation, and oxidative stress, to improve understanding of the pathophysiological mechanisms.
4. **Evaluation of behavioral intervention effects** (e.g., weight management programs, physical activity promotion, nutritional counseling, stress reduction) on preventing or slowing the development of endometrial lesions.
5. **Multidisciplinary approaches**, involving collaboration among gynecology, endocrinology, public health, and behavioral sciences, to develop sustainable prevention models and improve health literacy.
6. **Assessment of quality of life and psychological well-being** among women at elevated risk, to design interventions that not only reduce risk but also enhance physical and emotional health outcomes.

## Figures and Tables

**Table 1:** Baseline demographic and reproductive characteristics by diagnostic group.

Variable	Leiomyoma (n = 20)	EIN (n = 15)	EC (n = 15)	*p*-Value
**Age** (years), mean ± SD	50.0 ± 10.8	51.0 ± 9.5	56.1 ± 5.7	0.175
**BMI** (kg/m^2^), mean ± SD	26.3 ± 4.0	29.8 ± 4.6	29.4 ± 4.1	0.051
**Age at menarche** (years), mean ± SD	13.5 ± 1.3	13.2 ± 1.3	12.6 ± 1.3	0.178
**Menopause**, n (%)				
Yes	8 (40.0%)	8 (53.3%)	9 (60.0%)	0.480
No	12 (60.0%)	7 (46.7%)	6 (40.0%)	-
**Parity, median** (IQR)	2.0 (1.0–3.0)	2.0 (1.0–2.0)	1.0 (1.0–2.0)	0.855

**Table 2:** Lifestyle-related variables by diagnostic group.

Variable	Leiomyoma (n = 20)	EIN (n = 15)	EC (n = 15)	*p*-Value
**Physical activity**, n (%)				
Low (<75 min/week)	2 (10.0%)	4 (26.7%)	0 (0.0%)	0.018
Moderate (75–150 min/w)	14 (70.0%)	4 (26.7%)	6 (40.0%)	-
High (>150 min/w)	4 (20.0%)	7 (46.7%)	9 (60.0%)	-
**Smoking status**, n (%)				
Never	10 (50.0%)	7 (46.7%)	9 (60.0%)	0.408
Former	5 (25.0%)	1 (6.7%)	3 (20.0%)	-
Current	5 (25.0%)	7 (46.7%)	3 (20.0%)	-
**Alcohol intake** (units/week), mean ± SD	1.4 ± 1.4	2.0 ± 1.6	1.5 ± 1.6	0.566
**Sleep duration** (hours/night), mean ± SD	6.7 ± 0.9	7.6 ± 1.2	6.3 ± 1.1	0.021
**Perceived stress** (1–5), mean ± SD	3.0 ± 1.1	2.7 ± 1.0	3.0 ± 1.0	0.555
**Dietary index** (0–100), mean ± SD	59.4 ± 8.3	54.7 ± 11.3	58.3 ± 13.6	0.245

**Table 3:** (**a**) Univariate logistic regression for the association between lifestyle factors and EIN/EC vs. leiomyoma. (**b**) Multivariable logistic regression model including Lifestyle factors.

**(a)**		
**Predictor**	**OR (95% CI)**	***p*-Value**
**BMI** (kg/m^2^)	1.23 (1.04–1.45)	0.016
**Physical activity** (ordinal)	2.00 (0.82–4.86)	0.125
**Dietary index** (per 1 point)	0.98 (0.93–1.03)	0.357
**Sleep duration** (per 1 h)	1.17 (0.71–1.95)	0.533
**Perceived stress** (per 1 point)	0.81 (0.46–1.42)	0.463
**Smoking status** (ordinal)	1.07 (0.56–2.04)	0.844
**(b)**		
**Predictor**	**Adjusted OR (95% CI)**	***p*** **-Value**
**BMI** (kg/m^2^)	1.29 (1.06–1.58)	0.012
**Physical activity** (ordinal)	2.05 (0.72–5.83)	0.178
**Dietary index** (per 1 point)	0.94 (0.88–1.01)	0.101
**Sleep duration** (per 1 h)	1.52 (0.77–3.01)	0.230
**Perceived stress** (per 1 point)	0.75 (0.35–1.59)	0.454
**Smoking status** (ordinal)	1.48 (0.64–3.39)	0.359

**Table 4:** Distribution of metabolic, inflammatory, oxidative, and hormonal biomarkers in the subgroup with available laboratory data.

Biomarker	Category	Leiomyoma (n = 15)	EIN (n = 12)	EC (n = 11)	*p*-Value
**Insulin resistance**	Normal	12 (80.0%)	5 (41.7%)	7 (63.6%)	0.080
(e.g., insulin/HOMA-IR)	Elevated	3 (20.0%)	5 (41.7%)	6 (54.5%)	-
	Missing	0 (0.0%)	2 (16.7%)	0 (0.0%)	-
**Lipid profile**	Normal	11 (73.3%)	7 (58.3%)	6 (54.5%)	0.290
(LDL/HDL/TG)	Abnormal	3 (20.0%)	3 (25.0%)	4 (36.4%)	-
	Missing	1 (6.7%)	2 (16.7%)	1 (9.1%)	-
**hs-CRP**	Normal	13 (86.7%)	6 (50.0%)	7 (63.6%)	0.060
	Elevated	2 (13.3%)	4 (33.3%)	6 (54.5%)	-
	Missing	0 (0.0%)	2 (16.7%)	0 (0.0%)	-
**IL-6/TNF-α**	Normal	9 (60.0%)	6 (50.0%)	4 (36.4%)	0.040
	Elevated	0 (0.0%)	2 (16.7%)	3 (27.3%)	-
	Missing	6 (40.0%)	4 (33.3%)	4 (36.4%)	-
**Oxidative stress**	Normal	10 (66.7%)	7 (58.3%)	3 (27.3%)	0.030
(MDA)	Elevated	1 (6.7%)	2 (16.7%)	5 (45.5%)	-
	Missing	4 (26.7%)	3 (25.0%)	3 (27.3%)	-
**Hormonal profile**	Normal	9 (60.0%)	5 (41.7%)	3 (27.3%)	0.050
(E2/progesterone balance)	Elevated risk	3 (20.0%)	5 (41.7%)	6 (54.5%)	-
	Missing	3 (20.0%)	2 (16.7%)	2 (18.2%)	-

## Data Availability

The original contributions presented in this study are included in the article/Supplementary Materials. Further inquiries can be directed to the corresponding author as the project is still ongoing and not all data have been processed and published.

## Supplementary Materials

The following supporting information can be downloaded at: https://www.mdpi.com/article/10.3390/healthcare14020231/s1, Supplementary File S1: Lifestyle Risk Factor Questionnaire Used in the Study.
