# Supplementary material for: Lifestyle, Modifiable Behavioral Factors, and Biomarker Profiles in Uterine Lesions
Source: Healthcare (Basel). 2026 Jan 16;14(2):231. doi: 10.3390/healthcare14020231 (PMC12841236; doi:10.3390/healthcare14020231)
Supplement: Supplementary file 1 [file healthcare-14-00231-s001.zip › healthcare-4005578-supplementary.pdf]

Supplementary File S1. Lifestyle Risk Factor Questionnaire Used in the Study

Overview
This supplementary file provides the complete set of questionnaire items used to assess lifestyle-related risk factors among the study participants. The questionnaire was developed using domains derived from validated international instruments (IPAQ-SF, MEDAS, PSS, PSQI) but adapted for local clinical use. All items administered to participants are reproduced verbatim below.
Cultural adaptation included expert review by clinicians and public health specialists, followed by pilot testing to ensure clarity and contextual relevance for the target population.

Section 1. Personal and Anthropometric Data

- **Age:** ____ years
- **Height:** ____ cm
- **Weight:** ____ kg *(BMI calculated subsequently)*

Section 2. Menstrual and Reproductive History

- **Age at menarche:** ____ years
- **Menopause:**_Yes/No
  If yes → *Age at menopause:* ____ years
- **Number of births:** ____
- **Use of hormonal therapy or oral contraceptives:** Yes / No

Section 3. Lifestyle Factors
3.1 Smoking Status

- Never
- Former smoker
- Current smoker
  If current smoker → *Approximate number of cigarettes per day:* ____

3.2 Alcohol Consumption

- *Units per week:* ____

3.3 Physical Activity
Participants selected one of the following categories:

- **Low:** <75 minutes per week
- **Moderate:** 75–150 minutes per week
- **High:** >150 minutes per week

(Categories informed by IPAQ-SF guidelines.)
3.4 Sleep Duration

- *Hours of sleep per night:* ____

(Derived from the PSQI single-item sleep duration measure.)
3.5 Perceived Stress
Participants rated their level of subjective stress on a 5-point Likert scale:

- **Stress level:** 1 2 3 4 5
  (1 = low stress, 5 = high stress)

(Adapted from the conceptual framework of the Perceived Stress Scale.)
Section 4. Dietary Habits
4.1 Fruit and Vegetable Intake

- Rarely (≤2 times per week)
- Moderately (3–5 times per week)
- Daily

4.2 Red Meat and Processed Meat Consumption

- Frequently (≥4 times per week)
- Moderately (1–3 times per week)
- Rarely (≤1 time per week)

4.3 Consumption of Sugary Drinks

- Yes / No
  If yes → *Frequency:* ____

(Dietary items were mapped onto Mediterranean diet components and used to construct a simplified dietary index.)

Section 5. Family History of Cancer

- **Family history of endometrial, ovarian, or colorectal cancer:** Yes / No
  If yes → *Relationship and age at diagnosis:* ____________________

Scoring Procedures Used in the Study

Physical Activity:

Categorical variable (low / moderate / high).

Sleep Duration:

Continuous variable (hours/night).

Perceived Stress:

Single-item measure scored from 1 to 5 (continuous).

Dietary Index:

Composite score based on three items:

1. Frequency of fruit and vegetable consumption
2. Frequency of red/processed meat consumption
3. Sugary drink consumption

Higher scores reflected healthier dietary patterns.

Notes on Adaptation

- Items were adapted from validated instruments to suit local language, clinical feasibility, and patient literacy.
- Expert review ensured content validity.
- Pilot testing confirmed clarity and comprehension.
